# Efficacy and Threat Language in PFAS Messaging

**DOI:** 10.1111/risa.70239

**Published:** 2026-03-31

**Authors:** Rachel A. Hutchins, Lyn M. van Swol, Bret R. Shaw, Tim E. Holland, Gavin K. Dehnert

**Affiliations:** ^1^ Department of Communication Art University of Wisconsin–Madison Madison Wisconsin USA; ^2^ Department of Life Science Communication University of Wisconsin–Madison Madison Wisconsin USA; ^3^ Division of Extension University of Wisconsin–Madison Madison Wisconsin USA; ^4^ Nelson Institute for Environmental Studies University of Wisconsin–Madison Madison Wisconsin USA; ^5^ Sea Grant Institute University of Wisconsin Madison Wisconsin USA

**Keywords:** drinking water, environmental communication, Extended Parallel Process Model, health communication, municipal water, natural language processing, per‐ and polyfluoroalkyl substances, PFOA, PFOS, qualitative methodology, risk communication

## Abstract

As more Americans become aware of the threat of per‐ and polyfluoroalkyl substances (PFAS) in drinking water, they seek information using internet searches. Little is known, however, about the content of the websites produced by those searches or how well such content aligns with risk communication best practices. Guided by the Extended Parallel Processing Model and its two primary components, threat and efficacy, we analyze the top websites produced by Google searches relating to PFAS and water. We utilized a mixed‐methods approach, with both qualitative content analysis and computational linguistic analysis, to examine how threat and efficacy are conveyed and how these portrayals differ by the type of message source. Our results show that news media sources and research sources are more likely to emphasize threat severity and individual susceptibility as well as to use negative emotion and anxious language, particularly in comparison with local government sites or water utility boards. News media was also more likely to provide efficacy information than other sources, but the overall emphasis on efficacy was low. Our results highlight the difference between established best practices and reality, offering recommendations for communicators across all source types to improve their PFAS communication and better aid the public.

## 1 Introduction

Recently, the issue of per‐ and polyfluoroalkyl substances (PFAS) in drinking water has made front page news. For example, Erin Brockovich rang the alarm in a 2024 editorial in the *New York Times* (Brockovich 2024). As awareness increases around the threat of PFAS in drinking water, it is essential for communicators to understand what information people are encountering about PFAS, especially in online searches, to determine the best strategies for communicating this threat to the public.

PFAS, or forever chemicals, refer to a family of synthetic chemicals that have a long half‐life and break down slowly once they enter the human body (Harvard T.H. Chan School of Public Health 2018; National Wildlife Foundation n.d.). Research has connected them to a variety of negative health outcomes, such as cancer, liver damage, pregnancy complications, developmental delays in children, and reduced vaccine effectiveness (Fenton et al. 2021; Harvard T.H. Chan School of Public Health 2018; Hayes 2021). These health risks are particularly concerning because over 97% of Americans now have measurable levels of PFAS in their blood (Lewis et al. 2015; Pomazal et al. 2024). The most common source of PFAS exposure is drinking water (Post et al. 2017) after PFAS enters the water through industrial discharge, treated wastewater from which PFAS has not been removed, runoff from manufacturing sites, leaching from landfills, and other routes (Evans et al. 2020).

As people increasingly learn about PFAS in their drinking water, they are likely to begin information seeking with an internet search. Given the prominence of online searches for health and risk communication (Powell et al. 2011) and increasing interest in the role of information seeking in risk communication (Gutteling and de Vries 2017), it is important to understand what people are encountering when they search for information. PFAS communicators face a difficult balance; they must convey the threat of PFAS as well as provide efficacy information to help the public reduce their threat (Sellnow et al. 2008; Witte 1992), but this can be complicated for several reasons. First, the health risks of PFAS vary based on factors such as geographic location and the level of exposure (Post et al. 2017), and research in this area is ongoing and ever‐changing (Fenton et al. 2021). Efficacy recommendations similarly may vary depending on an individual's threat level. Communicators thus must determine how to convey the threat without downplaying it (Ducatman et al. 2022) or overstating it (Dempster et al. 2022), and they also must consider the public's ability to understand risk management strategies and scientific uncertainty (Fischhoff and David 2014). Jansen et al. (2020) found that people often struggle to interpret uncertain risks related to food additives and contaminants and may perceive the mere presence of an additive as disturbing, even if at low and safe levels. This tendency may similarly influence how the public interprets PFAS messaging, especially when uncertainty is emphasized. In addition, emphasis on threat and efficacy might vary depending on the source; government websites have different goals and face different constraints than news websites, for example (Harclerode et al. 2021). Yet, while juggling all these factors is a difficult task for communicators, it is critically important; if people searching for PFAS information are initially confronted with messages that don't strike the right balance, they will be less likely to continue seeking other information or take steps to reduce their PFAS exposure. It is therefore key to focus specifically on PFAS messaging that the information‐seeking public actually views rather than all available PFAS messaging (e.g., Ducatman et al. 2022; Zindel et al. 2021).

Thus, it is necessary to focus on the PFAS information the public is currently accessing online, examining how different types of sources portray the threat of PFAS exposure and the efficacy of taking action. We address this question by conducting a content analysis, using both human and computational coding, of the top websites accessed by Google users searching for information about PFAS in their drinking water. Using the Extended Parallel Process Model (EPPM) and its two primary constructs (threat and efficacy information) as our guiding theory, we examine how various website sources approach PFAS communication. We conclude by providing recommendations for improvement based on known best practices in risk communication in order to help all PFAS communicators create effective messaging.

### 1.1 Communicating PFAS

Effective PFAS communication is a growing concern at a time when many Americans are becoming aware of these chemicals for the first time (Berthold et al. 2023) and, thus, are likely to be conducting their first online searches about PFAS. Defining “effective PFAS communication” is complicated, however. Risk communication best practices highlight the importance of transparency (Seeger 2006), but at the same time, “risks are always associated with some level of uncertainty” (p. 239). In the context of PFAS, communicators must balance a need to honestly convey known threats, such as liver damage or reduced vaccine effectiveness (Fenton et al. 2021), while still acknowledging uncertainties such as the amount or length of PFAS exposure associated with these outcomes and differences in threat level by geographic location (Post et al. 2017) (A trick further complicated by people's tendency to focus on the mere presence of a chemical when weighing potential risks, rather than a deeper understanding of exposure and dosage [Kraus et al. 1992]). This aligns with broader findings on “chemophobia,” where consumers often equate the presence of any chemical with harm, regardless of dose or context (Saleh et al. 2019). Such perceptions can complicate PFAS communication by amplifying fear even when exposure levels are low. A few studies have examined the content of existing PFAS communication; for example, Ducatman et al. (2022) were critical of the depiction of risk in the governmental, nonprofit, and professional internet sources they reviewed, highlighting the “failure to distinguish levels of evidence for outcomes, with frequent emphasis on doubt that appears to inappropriately encompass all outcomes” (p. 5). The authors noted that many sources identified PFAS as a possible health threat but also used hedging language such as “may,” “some studies,” or “could.” Yet, as Harclerode et al. (2021) pointed out, the evolving science related to PFAS health impacts and the lack of guidance on standards for PFAS in drinking water, along with the difficulty in developing such standards (Post et al. 2017), complicate public health communicators’ and environmental agencies’ ability to provide firm recommendations. It also takes time for research findings, which often are not accompanied by press releases or media attention (Fuoco et al. 2023), to be incorporated into official sources and shared with the public. Even so, some organizations have called for sources to provide clearer information about the threat of PFAS exposure (National Academies of Sciences, Engineering, and Medicine 2022), especially as Americans become increasingly aware of these chemicals (Berthold et al. 2023) and use internet searches to find trusted sources for more information (Tian et al. 2022). When PFAS communicators must juggle transparency and acknowledgement of uncertainty (Seeger 2006), it is important to ensure that they do not rely too heavily on the latter at the expense of the former.

A second risk communication best practice that is relevant to PFAS communication is the need to take local context into account (Sellnow et al. 2008), both in terms of the different ways that people perceive the threat (Morgan et al. 2002) and the different levels of actual threat. This can be complicated when PFAS exposure in drinking water varies significantly by geographic location, and even neighboring cities could have vastly different levels of exposure due to point sources of contamination. Local sources may be better suited to address geographically specific questions about the threat (Harclerode et al. 2021), but these may not be the kinds of messages the public is most likely to encounter when searching online. Ducatman et al. (2022) also noted that existing PFAS communication often fails to address the needs of communities with high PFAS exposure, despite the fact that these communities are perhaps the most likely to seek information about PFAS (Berthold et al. 2023). Claassen et al. (2021) similarly emphasized that effective communication about emerging contaminants must be tailored to local concerns and information needs. Their study on GenX in the Netherlands found that addressing public uncertainty required not only transparency but also responsiveness to the specific questions and anxieties of affected communities.

A final risk communication best practice related to PFAS communication is self‐efficacy information. Efficacy messages provide information about specific actions people can take to address a threat, playing a key role in risk communication by providing people both with actionable steps to reduce the threat and with a sense of control amidst uncertainty (Seeger 2006). In the context of PFAS, Dong and Yang (2023) found that perceived self‐efficacy in addressing PFAS contamination was related to participants’ intention to take steps to protect themselves. Holland et al. (2025) found that efficacy‐related information—how to remove PFAS from drinking water—was the single largest category of questions people searched for on Google when looking for information about PFAS in drinking water supplies. However, existing PFAS communication seems to provide little efficacy information, despite calls to do so (National Academies of Sciences, Engineering, and Medicine 2022). In their review, Ducatman et al. (2022) noted that messaging was generally unlikely to provide information about how people can reduce the threat of PFAS exposure, particularly in high‐risk communities. Zindel et al. (2021) similarly highlighted a greater need for efficacy information on state PFAS websites. This lack may be because little is known about options to remove PFAS after exposure (Harclerode et al. 2021), leading to an emphasis on prevention rather than treatment (Anderko 2020). While there are some preventative actions that individuals can take in the context of drinking water, removing PFAS from a municipal water supply requires significant investment. If such investment has not yet occurred, official messaging may avoid discussing efficacy in this way or avoid providing official PFAS communication at all (Zindel et al. 2021).

Given these gaps in existing messaging, we utilize research and theory on risk and persuasion to provide guidance for improving PFAS communication. The EPPM (Witte 1992) provides a useful framework for crafting messages that convey the severity of the threat to the public while also providing actionable steps that individuals can take to reduce the threat and control their fear.

### 1.2 The Extended Parallel Process Model

The EPPM explores factors that persuade individuals to change their behavior, including fear of a threat and a sense of efficacy to enact a behavior to reduce the threat. The EPPM has its roots in health behaviors (Witte 1992), but the model has been applied in both health (Vos et al. 2018) and environmental (Hutchins et al. 2023) risk communication contexts. The model suggests that people must first perceive that a threat exists to consider changing behaviors to avoid the threat. The threat element of the EPPM includes two subcomponents: The individual must believe that the threat is severe (severity) and that they are personally susceptible to it (susceptibility). For example, individuals who have never heard of PFAS or who are unconvinced that PFAS pose threats to human health (low severity) are unlikely to care about the problem. Alternatively, individuals may know that PFAS is a threat to human health (high severity) but be unconcerned because they believe their own water supply is not affected (low susceptibility).

In addition, the EPPM argues that threat information by itself is not sufficient to persuade individuals to change their behavior. Indeed, overemphasis on fear can cause a persuasive backlash effect (Maloney et al. 2011). If people are made to feel fearful without a solution, they may prefer to avoid thinking about the threat or may deny the threat and may be dissuaded from attempting to address it (Munger 2019; Shen and Coles 2015). Rather, effective risk communication must include information about efficacy of solutions (Witte 1992). That is, individuals must believe that there are actions that will reduce the threat (response efficacy) and that they themselves are capable of doing these actions (self‐efficacy). For example, a message explaining that an expensive reverse osmosis system installed in one's house could reduce PFAS in drinking water may have high response efficacy, but individuals may not feel they have the self‐efficacy to enact this behavior. Thus, it is necessary to not only provide information about the threat of PFAS in drinking water but also inform individuals about feasible actions that can be taken to reduce this threat. Although the original conceptualization of efficacy in the EPPM focused on individual actions such as these, the model was later updated to take collective actions into account (collective efficacy; R. A. Smith et al. 2007). Collective efficacy messaging might focus on lobbying local or federal governments to better test, regulate, and treat PFAS in municipal water supplies rather than emphasizing individual actions.

Thus, both threat and efficacy play a key role in risk communication. The EPPM further suggests there are two primary outcomes to messages that incorporate these components. If, after exposure to a message, an individual's sense of threat outweighs their sense of efficacy, they engage in what the model calls fear control (Witte 1998). They might deny or downplay the threat, perhaps focusing on PFAS messages that convey doubt about risks to human health (Ducatman et al. 2022), or they might avoid thinking about the issue. With either of these approaches, this individual is unlikely to engage in steps to reduce the threat. That is, PFAS communication that overly emphasizes the threat without providing effective information about efficacy may backfire and lead to individuals not taking steps to protect themselves from PFAS contamination, as well as making them less receptive to further messages.

In contrast, if the individual's sense of efficacy outweighs their sense of threat and they feel empowered from the message to address the threat, they are more likely to engage in danger control, which involves changing their behavior and adopting a solution to reduce the threat (Witte 1998). PFAS messages that provide information about the threat but also inform the public about actions they can take to control it are more likely to persuade individuals to engage in those actions. For example, a PFAS message that emphasizes the ease and affordability of using a pitcher filter to reduce PFAS exposure could offer a high efficacy solution, leading to people engaging in danger control and using a pitcher filter to avoid PFAS exposure.

### 1.3 Message Sources

Various message sources may balance the threat and efficacy of PFAS differently, depending on their own goals and agenda, and so we also consider this aspect of messaging. The importance of source is well‐established in effective risk communication (Glik 2007), especially as people are often suspicious and less trusting of authority during stressful situations and, therefore, less trusting of official communication (R. G. Peters et al. 1997). Despite this, the limited research on PFAS communication tends to either combine all types of sources together (Ducatman et al. 2022) or look only at a single type of source (e.g., state government websites; Zindel et al. 2021) rather than considering how messaging may differ by source. The importance of this oversight becomes clear when examining PFAS communication through the lens of the EPPM; for this topic, as with many risk communication messages (Glik 2007), the message content might vary greatly depending on the intended audience or goals of the source. For example, the EPPM highlights the importance of conveying an individual's susceptibility to a given threat, but the audience's susceptibility to PFAS will vary depending on the audience. In a community with a high level of PFAS contamination, messaging from the local government may be more likely to discuss susceptibility than messaging from the federal government that is aimed at a national audience (or, conversely, federal government sources might find it easier to discuss susceptibility because of their focus on a broader geographic area). Similarly, information about self‐efficacy or collective efficacy will likely vary based on the goals of the source, such as nonprofits focusing on donations to fund their advocacy work to promote collective action steps. Communication of threat severity could also vary by source. Whereas local governments may not want to alarm the public, nonprofits or news media sources could use high severity messages as a way to attract an audience to their message and create a sense of urgency to get involved with the issue. As these examples demonstrate, it is critically important for risk communication to examine the message source based on their target audience and the message itself (Gutteling and Wiegman 1996).

Message sources may also differ in the specific language they use to discuss PFAS, such as the extent to which they use negative or fearful language. This may trigger the affect heuristic in readers and influence evaluations of these chemicals. The affect heuristic refers to a decision‐making strategy in which individuals rely on feelings or emotional responses rather than objective information (Slovic et al. 2007). This heuristic has also been applied to risk perceptions; when individuals read a message about PFAS, for example, they are likely to draw on their existing perceptions of related risks and benefits as well as their affective evaluation of those risks and benefits (Finucane et al. 2000). Yet these affective evaluations can be influenced through language use (Slovic et al. 2007). Threat can be conveyed with statistical probabilities but is also commonly associated with language related to negative emotion (E. M. Peters et al. 2004) and fear/worry (Griffin et al. 1999). Some message sources might capitalize on those emotions to better highlight PFAS as a threat, while others may be more likely to avoid such affective language. These affective evaluations may be especially pronounced in the context of chemical risks. Saleh et al. (2019) found that many consumers exhibit “chemophobia,” a tendency to view all chemicals as inherently harmful regardless of context or dose. This mindset may amplify the emotional impact of PFAS messaging, particularly when threat is emphasized without sufficient nuance. Conversely, efficacy is associated with positive emotions such as hope (Chen and Chen 2023; Nabi and Myrick 2019). Sources emphasizing efficacy might then be more likely to use positive language than sources focused primarily on the threat itself.

In this study, we apply the EPPM to existing messaging across source types to assess the current state of PFAS communication and provide recommendations for improvement. We ask:

*RQ: How does PFAS messaging about efficacy and threat differ by message source?*

## 2 Methods

### 2.1 Sample

Using the search engine analytics tool Semrush, we extracted a list of the top 100 websites (see Appendix A) accessed by Google users when searches include two keywords: “PFAS” and “water” (e.g., “how does PFAS get into water”, “how to remove PFAS from water”, and “what cities have PFAS in drinking water”). Semrush is an online platform that connects to Google Analytics to allow users to run search engine optimization and other tools. Its ranking feature provides users with information about the domains receiving the highest traffic from a given search. Semrush utilizes a proprietary algorithm and third‐party data providers to extract Google search analytics, which are updated monthly in Semrush (Semrush, n.d.). Since the AI algorithm is unknown, research cannot define the levels of accuracy or bias within the website for prioritizing certain websites. However, businesses that utilize Semrush for marketing analytics consider it to be largely accurate and representative of Google Search Engine Results Page (SERP) rankings. For this research, the results are likely representative of what Google users view when searches include “PFAS” and “water” as keywords. The use of Semrush provides quantitative insights into how people search for information online about PFAS in water and which websites they visit as a result of those searches. We utilized Semrush in November 2023 to extract the 100 most‐accessed websites, but the list included 104 websites as there were several ties in ranks. Three sources were removed from the list because they were duplicates or the website was no longer accessible. An additional three sources coded as corporate (see Section 2.2) were removed due to the small cell size, resulting in a final sample of 98 websites. These 98 websites identified with SEMrush were then coded by both human coders and with the software coding program Linguistic Inquiry and Word Count (LIWC).

### 2.2 Measures

#### 2.2.1 Threat Severity, Susceptibility, Individual Efficacy, and Collective Efficacy

Research assistants reviewed the main page of each website and rated each variable on a scale of 1–7. Threat severity measured the degree to which the source emphasized the risk of PFAS to human health (1 = *not at all risky*; 7 = *very risky*). Susceptibility measured the degree to which the source emphasized people's individual susceptibility to those risks (1 = *not at all susceptible*; 7 = *very susceptible*). For individual efficacy, coders examined whether the source clearly provided individual actions people can take to address PFAS (1 = *not at all*; 7 = *extensive information*). Finally, coders rated collective efficacy by noting the degree to which the source clearly provided information about collective actions being taken to reduce PFAS exposure (1 = *no information*; 7 = *extensive information*).

### 2.3 Procedure

Websites were analyzed using two separate methods: Human coding and then software coding. The first author categorized each website by type, including federal government (11.88%), state government (37.62%), local government (13.86%), nonprofit (10.89%), water utility (10.89%), news media (6.94%), research (5.94%), and corporate (2.97%—excluded from the analysis due to the small cell size). Two research assistants independently conducted a content analysis of each website, coding for the four of the elements of the EPPM (threat severity, susceptibility, individual efficacy, and collective efficacy; Krippendorf's alpha = 0.73). Coders were trained with examples of each concept. Approximately 10 percent of websites were coded independently by the first author and the two research assistants and then discussed to reach consensus. The remaining websites were then coded by the research assistants, using the codebook examples and discussion as a guide. We limited our analysis to content from the page that initially loaded when we accessed each webpage, excluding any drop‐down menus, links, or other content that requires users to engage with the page in order to access that information. The main pages of all websites were coded by each research assistant.

Such content analysis with human coders is the traditional approach, but messages can also be coded through computer software that can categorize the linguistic features of messages that relate to their portrayal of threat and efficacy. Investigating these linguistic markers of threat and efficacy using natural language processing provides an opportunity for researchers to capitalize on the strengths of each approach (Johnson and Onwuegbuzie 2004) and enhance the validity of the results through triangulation of multiple methods (Creswell and Creswell 2022). Given this, we examine our research question using both traditional content analysis with human coders and computational language analysis.

For the computational linguistic analysis, we analyzed the text of each website using the most current version of the LIWC (Boyd et al. 2022). The LIWC is a linguistic analysis software that provides the percentage of words in a given text that match a particular category. Most LIWC output variables represent the proportion of words in a given text that fall into specific psychological or linguistic categories, such as positive emotion. For instance, if five out of every 100 words in a transcript are positive emotions, the LIWC score for that category would be 5%. The software works by comparing the words in the analyzed text to those stored in its internal dictionaries. Since its initial development by Francis and Pennebaker in 1993, LIWC's dictionaries have undergone four major updates (Boyd et al. 2022; Pennebaker et al. 2001, 2007, 2015). The most recent version, LIWC‐22, includes over 12,000 terms organized into 117 psychologically meaningful categories, such as assent words, emotional expressions, cognitive mechanisms, and more (see Boyd et al. 2022, 5, 11, 12 for details).

We utilized three LIWC variables in our analysis: Positive emotion, negative emotion, and references to anxiety. Table 1 shows a summary of each LIWC category analyzed along with the relevant EPPM construct^1^. Other existing LIWC dictionaries do not align well with our focus on threat and efficacy, but it is also possible to build new dictionaries to measure constructs that existing dictionaries cannot capture; for example, validating a domain‐general dictionary for the concept of efficacy would be difficult because language related to efficacy is likely to either be too broad (e.g., can, do, act) or too specific (e.g., reverse osmosis, filter). Creating a PFAS‐specific efficacy dictionary, however, would allow researchers to examine efficacy in this specific context in a more cost‐effective and efficient way than through traditional content analysis. Therefore, we developed a new LIWC dictionary after consulting with water contaminant specialists and using Semrush data to identify commonly used efficacy words related to PFAS and water (Table 2), such as filter, install, test, treat, reverse osmosis, bottle, and avoid.

**TABLE 1:** Linguistic inquiry and word count (LIWC) categories and examples

Category name	LIWC example dictionary words	Related EPPM construct	Example
Positive emotion	Good, love, happy, hope	Efficacy	“Absolute power: Conquer your PFAS challenges. No matter your PFAS challenge, [company name] will **strengthen** your analytical game plan” (Source #54).
Negative emotion	Bad, hate, hurt, tired	Threat	“It was kinda like a slow, rolling **nightmare**” (Source #102).
Anxiety	Worry, fear, afraid, nervous	Threat	“There's nothing that will magically fix this…it looks **frightening**” (Source #28).
PFAS Efficacy	Filter, test, bottle, reverse osmosis	Efficacy	“There are two main types of water **treatment** systems that work best at **removing** PFAS from drinking water in people's homes” (Souce #50).

*Note*: See full data set for each source.

**TABLE 2:** PFAS efficacy dictionary.

Words included
Filter^a^
Test^a^
Limit^a^
Treat^a^
Reduc^a^
Identif^a^
Reverse osmosis
Effectiv^a^
Assess^a^
Bottl^a^
Remov^a^

^a^Informs the LIWC to include all words that use the provided stem. For example, “filter^a^” would capture words including filter, filtering, filtered, etc.

#### 2.3.1 Positive Emotion, Negative Emotion, Anxiety, and PFAS Efficacy

Linguistic analysis using the LIWC (Boyd et al. 2022) provided values for each source for each of the four dictionaries we selected: Positive emotion, negative emotion, anxiety, and the PFAS Efficacy dictionary we created (Table 2). Each value corresponded to the percentage of words in a text that matched the selected category, from 0 to 100.

### 2.4 Data Analysis

We conducted correlation analysis as well as either ANOVA or Kruskal–Wallis tests to compare message sources. We first used correlation analysis to compare the variables from the qualitative coding (threat severity, susceptibility, individual efficacy, and collective efficacy) and the variables from the linguistic analysis (positive emotion, negative emotion, anxiety, and PFAS efficacy) in order to determine whether the relationships matched those we expected (Table 1). We used Stata version 17.0 to calculate Pearson's correlation coefficient for each pair of variables. Second, we analyzed differences among message sources for each variable. ANOVA was used for the content analysis variables, but we selected Kruskal–Wallis *H* tests for the linguistic analysis variables because LIWC variables are seldom normally distributed.

## 3 Results

### 3.1 Descriptive Statistics

We begin by presenting the descriptive results for each variable, by source type (Table 3). As Table 3 demonstrates, the unstandardized means were very small in some cases, particularly for LIWC variables; this suggests that certain source types included little or no content that fell into this category.

**TABLE 3:** Unstandardized means^2^ by source type.

	Scale	Federal	State	Local	Water utility	Nonprofit	News media	Research
Risk	1–7	2.96	2.78	2.32	2	3.14	4.33	4.17
Susceptibility	1–7	3.83	3.49	2.68	2.91	3.59	5.17	4.58
Coder‐rated self‐efficacy	1–7	1.29	2.05	1.21	2.5	1.91	3.75	2.67
Collective efficacy	1–7	3.33	3.26	3.89	3.64	4.23	4.5	4.17
Positive emotion	0%–100%	0.7%	4%	4%	1%	3%	13%	4%
Negative emotion	0%–100%	0.4%	4%	2%	5%	7%	25%	3%
Anxiety	0%–100%	0.4%	1%	0%	2%	3%	19%	3%
LIWC efficacy	0%–100%	1.10%	1.25%	1.59%	1.99%	1.49%	1.96%	1.96%

### 3.2 Severity and Susceptibility

We examined results related to the EPPM categories of threat severity and susceptibility, beginning with correlation analysis. We found that human coder perceptions of severity and susceptibility were highly correlated (*r* = 0.683, *p* < 0.001), indicating that sources emphasizing the risk of PFAS to human health were also likely to highlight people's individual susceptibility to those risks. We also found that severity and susceptibility each had a significant positive correlation with the LIWC negative emotion variable (severity: *r* = 0.226, *p* = 0.026; susceptibility: *r* = 0.253, *p* = 0.012) and with the LIWC anxiety variable (severity: *r* = 0.239, *p* = 0.018; susceptibility: *r* = 0.211, *p* = 0.036). These correlations between coder perceptions of severity or susceptibility and the emotion variables from computational analysis demonstrate the connection between the two aspects of our analysis and the value of our mixed‐methods approach.

Next, we conducted one‐way ANOVAs to determine whether threat severity and susceptibility differed by message source. There was a significant difference in severity across message sources, *F*(6, 91) = 3.79, *p* = 0.002, *η* ^2^ = 0.20. We conducted post‐hoc tests using Tukey–Kramer due to unequal cell sizes and found significant differences between news media or research sources as compared to local governments and water utilities. As shown in Table 4, news sources were more likely to highlight the severity of PFAS contamination than local governments (1.38 ± 0.43, *p* = 0.029). Similarly, water utility websites were less likely to focus on severity than news sources (−1.61 ± 0.49, *p* = 0.009). Water utility websites were also less likely to emphasize severity than research sources (−1.52 ± 0.45, *p* = 0.020).

**TABLE 4:** Source differences by variable.

Variable	Low	High
Severity	Local government*	News media*
	Water utility boards*°	Research°
Susceptibility	Local government*	News media*
	Water utility boards*	
Negative emotion	Federal government *	News media*
	State government *	
	Local government *	
Anxiety words	Federal government*	News media*
	State government*°	Nonprofit°
	Local government*°	
	Water utility boards*	
Collective efficacy	—	—
Coder‐rated self‐efficacy	Federal government*	News media*
	Local government*	
Positive emotion	Local government*	News media*
	Water utility boards*	
	Federal government*	
	State government*	
	Nonprofit*	
LIWC efficacy	—	—

*Note*: Symbols indicate significant pair‐wise comparisons for each category. For example, an asterisk next to a message source in the center column indicates that source was significantly different from the asterisked source in the right‐hand column. Dashes indicate no statistically significant differences by source type.

The level of susceptibility also differed significantly by website source, *F*(6, 91) = 3.06, *p* = 0.009, *η* ^2^ = 0.17. Tukey post‐hoc tests again revealed similar trends. We found a significant difference, with news media emphasizing susceptibility more compared to local governments (1.48 ± 0.43, *p* = 0.013). There was also a significant difference between water utility websites compared to news media (−1.34 ± 0.44, *p* = 0.049). News media is significantly more likely to emphasize individuals’ susceptibility to PFAS contamination in their drinking water than local sources such as water utilities and local governments.

Based on the finding that local sources were particularly unlikely to discuss susceptibility to PFAS exposure, we conducted additional exploratory analysis. Using just the 25 sources we coded as either local government or municipal water utilities, two research assistants compiled a list of PFAS test results from the geographic location of each source (unstandardized mean PFAS level = 13.56 parts per trillion). For each location, we obtained between PFAS values from various testing sites within that geographic area. When locations had multiple testing sites or results (up to five), we utilized the largest value. We then used linear regression to regress the local areas’ actual PFAS levels onto susceptibility. We wanted to test if local areas that tested higher for PFAS levels would use more susceptibility language on their website, but the result was not statistically significant (*β* = −0.01, *p* = 0.361). Thus, for local government and water utility sources, there was no relationship between levels of PFAS found in their area and discussion of susceptibility to PFAS on their website.

Next, we turned to computational linguistic analysis using LIWC variables related to severity and susceptibility, including negative emotion and references to anxiety. We conducted Kruskal–Wallis *H* tests and found statistically significant differences among message sources for both of our variables: Negative emotion (*χ* ^2^[6] = 19.76, *p* = 0.0003) and references to anxiety (*χ* ^2^[6] = 25.81, *p* = 0.0002). Conover–Iman post‐hoc tests with Bonferroni correction showed that news media sources used a higher percentage of negative emotion words than federal, local, or state government sources (*p* < 0.018; Table 4). News media also used more anxious language than those same three sources as well as water utility boards (*p* < 0.02). Nonprofit sources also had a higher percentage of anxiety‐related words than local or state government websites (*p* < 0.02).

In sum, these results suggested that news media is more likely to emphasize the threat of PFAS contamination, including both overall severity and individual susceptibility, than other sources. Local government and water utility sources were particularly unlikely to highlight severity and susceptibility, and this finding held true regardless of the actual PFAS levels measured in that geographic area. The linguistic analysis results also differentiated news media sources from other types of sources, with news media more likely to use negative and fearful emotional language when discussing PFAS.

### 3.3 Self‐Efficacy and Collective Efficacy

Next, we examined both qualitative and linguistic analysis results related to self‐efficacy and collective efficacy, in line with the second half of the EPPM model (R. A. Smith et al. 2007). We again began with a correlation analysis. Coder‐rated efficacy of the website had a significant positive correlation with the LIWC efficacy variable we created (*r* = 0.49, *p* < 0.001). Positive emotion, contrary to our expectation, was not significantly correlated with coder‐rated efficacy (*r* = 0.10, *p* = 0.333); in fact, it was not significantly correlated with any of our variables. Interestingly, collective efficacy was also not significantly correlated with either the human‐coded efficacy variable (*r* = −0.01, *p* = 0.890) or our LIWC efficacy variable (*r* = 0.10, *p* = 0.326). It was, however, correlated with both severity (*r* = 0.29, *p* = 0.004) and susceptibility (*r* = 0.30, *p* = 0.003), although these correlations were relatively small.

Next, we conducted one‐way ANOVAs to examine differences in self‐efficacy and collective efficacy by source type. There was no significant difference among the message sources for collective efficacy, *F*(6, 91) = 1.87, *p* = .093). We did find a significant effect for self‐efficacy, *F*(6, 91) = 2.63, *p* = 0.022, *η* ^2^ = 0.15. As with the severity and susceptibility results, Tukey post‐hoc analysis again found a significant difference between news media sources and local government sources (1.52 ± 0.46, *p* = 0.022; Table 4). News media was also significantly more likely to emphasize self‐efficacy compared to federal government sources (1.47 ± 0.47, *p* = 0.038). It is worth noting, however, that the unstandardized mean for self‐efficacy across our sample was 2.02 on a scale of 1–7, suggesting all types of websites provided relatively little information about actions individuals can take to reduce the threat of PFAS exposure.

We also used linguistic analysis to examine source differences based on LIWC variables, including positive emotion and the LIWC efficacy dictionary we created. We again used a Kruskal–Wallis *H* test and found that both variables were statistically significant (positive emotion *χ* ^2^[6] = 21.46, *p* = 0.002; LIWC efficacy: *χ* ^2^[6] = 13.73, *p* = 0.033). Conover–Iman post‐hoc tests with Bonferroni correction showed that positive emotion was significantly higher for news media sources compared to federal, state, and local government as well as nonprofit and water utility sources (*p* < 0.005). For the LIWC efficacy variables, however, post‐hoc tests found no statistically significant pair‐wise comparisons.

## 4 Discussion

Since online searches are often the first place people seek health‐ and risk‐related information (Powell et al. 2011), we examined the top 98 websites accessed by Google users in the United States searching for information about PFAS in their drinking water. Through content analysis and computational linguistic analysis, we explored the ways in which the content of PFAS messaging differed by message source and applied the lens of the EPPM to consider how these websites balanced their appeals to threat and efficacy. Two themes emerged from analyses: Differentiating news media from local sources in their emphasis on the overall severity of PFAS exposure and individual susceptibility to this threat, and revealing similarities across all sources in how they talk about efficacy and in the general lack of efficacy information.

First, many of our results highlighted differences between news media and other types of sources. News media, compared to local government and water utility boards, was significantly more likely to emphasize the threat of PFAS, including both severity and individual susceptibility, as well as to use negative and fearful emotional language. We offer two possible explanations for these differences. One is the tendency for journalistic sources to use a different style of writing compared to that used on government and other websites, demonstrating a degree of emotional sensationalism in news sources (Brown et al. 2018) and suggesting that they are overemphasizing the threat of PFAS. The results of our linguistic analysis somewhat support this explanation, especially given our focus on online search results. In order for a particular news media article to be included in the top Google search results, it may have attracted more attention and clicks with more emotional and fearful language regarding the threat of PFAS.

A second explanation, however, is that these news sources are more accurately reporting the threat of PFAS contamination than, for example, government sources, who are likely cautious about emphasizing the threat or using negative emotional language to discuss it (Reynolds 2011). Supporting this explanation is our finding that research sources, like news sources, were also more likely to emphasize severity than other sources such as water utility boards. Research on PFAS in recent years has increasingly provided evidence of the threat and the negative impacts on human health (Fenton et al. 2021), and since many of the news sources in our list of websites were reporting on recent studies, both news websites and research websites had more emphasis on threat severity. For example, one news report wrote, “The study showed a link between 14 different PFAS chemicals and cancer, birth defects, thyroid disease, and liver damage. Other studies on PFAS have linked consumption to high cholesterol and nerve disorders” (Source #26). Thus, it is possible that the level of threat severity reported in these sources accurately reflects the threat that PFAS pose to human health and that it is the local government sites and water utility sites that are downplaying the threat. While health and science journalists are sometimes critiqued for inaccurately portraying research results (Dempster et al. 2022), our results suggest a different trend for PFAS messaging. At the same time, however, our finding that government websites were less likely to emphasize severity might stem in part from the complexity of their role in this situation; while public agencies aim to inform the public, they are also more likely to face financial and regulatory pressures related to addressing PFAS contamination (Harclerode et al. 2021) than media or research sources, making them perhaps more cautious about how they portray the threat. Some researchers have critiqued official communications for deemphasizing or downplaying the health risks of PFAS exposure (Ducatman et al. 2022; Zindel et al. 2021) and while our results offer a similar interpretation, it is also true that journalists and researchers typically have less oversight regarding their messaging than official communicators (Amend and Secko 2012). They might have more freedom to focus on threat severity as well as a goal to fulfill the journalistic “watchdog” role.

Many of the sources in our sample—except news media and research sources—seem to focus on uncertainty related to PFAS rather than on the threat itself, perhaps in an attempt to avoid overwhelming the public. Yet, while not scaring the public seems a worthwhile goal, existing research on risk communication (Aguirre 2005; Norwood 2005) and fear appeals (Tannenbaum et al. 2015) refutes the stereotype that people will panic when faced with a significant threat. Rather, it is necessary to provide sufficient and accurate information about the threat so as to educate people that the threat exists. Instead, many of the sources in our sample appeared to hedge when discussing the risks of PFAS exposure on human health, such as the federal government source that wrote, “scientific studies have shown that exposure to *some* [emphasis added] PFAS in the environment *may* be linked to harmful health effects in humans and animals” (Source #1). This may again relate to the communication goals of different sources as well as the underlying pressures they face, but at the same time, risk communication best practices emphasize the importance of transparency regarding both the threat and the related uncertainties (Seeger 2006). Writing about the communication of scientific uncertainty, Fischoff and Davis (2014) wrote, “communications are effective when they help people identify choices that serve their own, self‐defined best interests…with non‐persuasive (communications), honesty is the only policy” (p. 13664). Since good risk communication addresses known risks while still acknowledging existing uncertainties (Seeger 2006), our first recommendation to PFAS communicators is to ensure that their messaging succinctly provides information about known threats using straightforward language and separately provides information about areas in which PFAS research is ongoing.

In addition to differentiating news media (and, to a lesser extent, research sources) based on their emphasis on threat severity, our results also highlighted the differences between news media and other message sources in the degree to which they discussed people's susceptibility to PFAS. On one level, this is unsurprising. The news media sources included in our sample tended to be national news sites with a broad audience, and so their discussions of susceptibility were not focused on a particular geographic region but instead tended to emphasize the ubiquity of PFAS contamination and highlight its prevalence across the United States. For example, one news article wrote, “Almost half of the tap water in the United States is contaminated with chemicals known as ‘forever chemicals’ …the number of people drinking contaminated water may be even higher than what the study found” (Source #28). In contrast, local government websites and water utility sites focused on a specific geographic location, making them able to report the levels of PFAS found in a sample from a specific location with more precision. For example, one water utility source wrote, “DC Water's current test results from samples collected over the past two years are below the Maximum Contaminant Levels (MCLs) and Hazard Index” (Source #59). However, as our results showed, local sources’ potential ability to discuss susceptibility does not always correlate with an increased likelihood of doing so. These differences may again reflect the goals of the different sources. News media likely wants to attract attention and headlines (Brown et al. 2018; Dempster et al. 2022), and suggesting their audience is susceptible to the health effects of a dangerous chemical would suit that goal. Some news sources also focused on susceptibility in certain locations in the context of telling a human‐interest story about individuals affected by PFAS exposure in that location, an approach that fits the journalistic goal of providing a personal note to a science story (Amend and Secko 2012). In contrast, local governments and water utility boards want to reassure their constituents (Ryan 2021) rather than scare them or call attention to PFAS exposure in the area, but they may also be required to report certain PFAS levels in a straightforward manner or be facing the challenge of cleaning up a contaminated area.

However, given the variability in PFAS contamination at the local level, we might expect to see variability in the local sources’ discussions of susceptibility. For example, Zindel et al. (2021) found that states with higher PFAS levels were more likely to have a webpage about PFAS than states with lower levels. Instead, our analysis of local sources demonstrated that they were generally unlikely to discuss susceptibility to PFAS contamination, even if their audience was highly susceptible due to documented PFAS in the area's drinking water. For example, one local government source with high PFAS levels at multiple test sites said nothing about PFAS levels in the area and instead directed readers to “view our 2024 Water Quality Report…for information about your drinking water source” (Source #60), a report that directed readers to yet another report.^3^ While it is useful for messaging to reassure people who are not actually at risk for PFAS communication (Sellnow et al. 2008), it is particularly important that susceptible individuals are made aware of their risk. This is even more true given that people often underestimate their risk (Tversky and Kahneman 1986), especially in situations where messaging highlights doubts about the risk or includes extensive scientific or technical information (Slovic et al. 1981). Given these findings, our second recommendation is that PFAS communicators accurately depict their audience's level of susceptibility in order to ensure that their messaging is efficacious for that community (Ducatman et al. 2022).

The second theme emerging from our results was the overall lack of efficacy information found across all types of sources. While we found no significant differences across types of sources in terms of collective efficacy or in terms of percentage of efficacy words as measured by our LIWC dictionary, our human coding results did show significant pair‐wise differences in self‐efficacy, differentiating news media sources as high‐efficacy but local and federal government sources as low efficacy. This demonstrates an attempt by news sources to balance their emphasis on severity and susceptibility with the efficacy information that good risk communication requires (Seeger 2006; Witte 1992) and PFAS messaging often lacks (Ducatman et al. 2022; Zindel et al. 2021). However, the unstandardized mean for individual efficacy coded by our research assistants was quite low: 2.02 out of 7 (Appendix B). This may have been a result of our coding approach; although we analyzed only the content that was visible when a page initially loaded, we did code a source as a 2 if the page included a link to further information about that topic. Many sources did not include efficacy information on the primary page but did include a link about efficacy, such as the state government source with links reading, “How can I reduce my exposure?” and “How do I test my water for PFAS?” (Source #20). Without access to click‐through ratios, we cannot tell for certain whether most people would utilize the available links and access the efficacy information provided. Even so, tests of the EPPM traditionally combine threat and efficacy information in a single message (S. W. Smith et al. 2008) in order to ensure the individual is exposed to the relevant information. Given this, we strongly urge PFAS communicators to highlight efficacy information along with threat information, as well as including more detailed efficacy information elsewhere. Providing specific information about individual or collective actions can increase the likelihood that they engage in the recommended behaviors (Tierney 2000).

Surprisingly, we found no significant correlation between positive emotion and either self‐ or collective efficacy. In contrast, other studies have found associations between feelings of self‐efficacy and positive emotions such as hope (Nabi and Myrick 2019), theorizing that the fear created by a threatening message can be eased by a sense of hope or relief by discovering that actionable steps exist (Nabi 2015). However, this approach focuses on the perception of fear or hope in the individual exposed to the message, rather than the expression of these emotions in the message itself. It is possible that positive language in PFAS messaging has the potential to improve message acceptance or promote behavioral changes if measured correctly (e.g., hopeful language rather than positive emotion generally); future research could explore this. We did, however, find significant differences in the use of positive emotion in different sources, with news media again more likely to use positive emotional language than most other source types. The lack of correlations with our efficacy measures suggests that this finding is less related to efficacy, though, and instead likely stems from the journalistic goals discussed above, such as utilizing a human‐interest angle for a risk communication story (Amend and Secko 2012).

Finally, this paper adds to the literature on risk communication with PFAS by using a computational approach and creating a dictionary of PFAS efficacy terms. For researchers using larger datasets of PFAS websites, a computational approach allows categorizing communications quickly and efficiently. Further, using a consistent computational approach allows for comparison across studies.

### 4.1 Limitations

There are several limitations to our study. First, we only accessed the URLs of the most‐accessed sources on a national level. We had no information about users’ geographic location or other demographic information that might have added nuance to these results. We also were unable to assess people's responses to the websites they viewed, including changes in their knowledge, perception of the threat, or behavioral outcomes. Further research should examine how messaging factors such as susceptibility, severity, and efficacy influence individuals’ perceptions of and behaviors related to PFAS contamination. Second, as mentioned previously, we analyzed only the landing page for each source, rather than including information from drop‐down menus or other links that people viewing the website may have clicked through. It is also worth noting that while we found differences between source types for many of our variables, the unstandardized means for some variables were quite low (particularly for LIWC variables such as positive or negative emotion). Finally, our results were based on the top accessed websites in late 2023, but as the issue of PFAS gains more prominence, information on the websites people encounter in online searches is certainly going to change. We also accessed only the top websites as viewed by individuals in the United States, but we acknowledge that findings might differ greatly in other geographic locations, other regulatory contexts, or when using Semrush to focus on a larger or smaller scale.

### 4.2 Future Research

Given the results of our study, we argue that the EPPM and its two primary constructs (threat and efficacy) are relevant in the context of PFAS. Future research should consider empirically testing this assertion. We encourage researchers to consider testing whether information about severity, susceptibility, or efficacy in the context of PFAS has an effect on people's attitudes toward PFAS or on their protective behaviors. Future research might also extend on our findings about differences by messages source and explore how the attitudinal or behavioral effects of these messages differ depending on the source. Finally, our study focused on message sources’ content as of November 2023; future research could consider examining how this content changes over time and how the public's knowledge of PFAS relates to such changes.

## 5 Conclusion

Through content analysis and computational linguistic analysis, we have demonstrated that PFAS messaging differs by source type and that communication about both the threat itself and about actions that can be taken in response to the threat differ. At a time when online searches are a common form of information seeking (Powell et al. 2011) and awareness about PFAS exposure is growing (Berthold et al. 2023), it is important for PFAS communicators to assess whether commonly‐accessed websites are accurately portraying the risks of PFAS exposure, people's susceptibility to such exposure, and both individual and collective actions that can be taken to reduce the risk. Our results and recommendations provide a potential path forward for PFAS communicators. As public awareness of PFAS grows, now is the time to improve PFAS messaging to better meet the needs of all Americans.

## Conflicts of Interest

The authors declare no conflicts of interest.

## Supporting information




**Supplementary Information: Table B.1**: Unstandardized Means and Standard Deviations by Source Type

## Endnotes

1 The complete dataset and PFAS efficacy dictionary are available at https://osf.io/zhkfa/?view_only = 95b661ec8b3b4050b708998d3c7ce4c6

2 Note that the values in Table 3 are means. Values may not match reported results because the Kruskal–Wallis test is a non‐parametric test utilizing medians rather than means.

3 Source #60 directed readers to a report concluding that PFAS levels were below reportable levels. However, our research assistants found additional testing from a report by the Environmental Working Group in which PFAS levels at multiple sites were above reportable levels (4 ppt [parts per trillion]), including several above 35 ppt.
