# Supplementary material for: Efficacy and Threat Language in PFAS Messaging
Source: Risk Anal. 2026 Mar 31;46(4):e70239. doi: 10.1111/risa.70239 (PMC13040124; doi:10.1111/risa.70239)
Supplement: Supplementary file 1 — Supplementary Information: Table B.1: Unstandardized Means and Standard Deviations by Source Type [file RISA-46-0-s001.docx]

**1. APPENDIX A: List of Sources**

| **ID** | **Rank** | **Website** | **URL** |
| --- | --- | --- | --- |
| 1 | 1 | Environmental Protection Agency | <https://www.epa.gov/pfas/pfas-explained> |
| 2 | 1 | Vermont Department of Health | <https://www.healthvermont.gov/environment/drinking-water/perfluoroalkyl-and-polyfluoroalkyl-substances-pfas-drinking-water> |
| 3 | 1 | Illinois Department of Public Health | <https://dph.illinois.gov/topics-services/environmental-health-protection/private-water/fact-sheets/pfas-drinking-water.html> |
| 4 | 1 | Pretty Organic Girl | <https://www.prettyorganicgirl.com/water> |
| 5 | 1 | New York Department of Health | <https://www.health.ny.gov/environmental/investigations/drinkingwaterresponse/docs/atsdr_pfas_factsheet.pdf> |
| 6 | 2 | US Geological Survey | <https://www.usgs.gov/news/national-news-release/tap-water-study-detects-pfas-forever-chemicals-across-us> |
| 7 | 3 | Environmental Protection Agency | <https://www.epa.gov/pfas> |
| 8 | 4 | Illinois Department of Health | <https://dph.illinois.gov/topics-services/environmental-health-protection/private-water/fact-sheets/pfas-drinking-water.html> |
| 9 | 5 | Rhode Island Department of Health | https://health.ri.gov/water/about/pfas/ |
| 10 | 6 | Environmental Working Group | https://www.ewg.org/interactive-maps/pfas_contamination/ |
| 11 | 7 | Center for Disease Control and Prevention | <https://www.cdc.gov/biomonitoring/PFAS_FactSheet.html> |
| 12 | 8 | American Association for the Advancement of Science | <https://www.aaas.org/epi-center/pfas> |
| 13 | 9 | California Water Resources Control Board | https://www.waterboards.ca.gov/pfas/ |
| 14 | 10 | Minnesota Pollution Control Agency | <https://www.pca.state.mn.us/pollutants-and-contaminants/pfas> |
| 15 | 11 | NIH Environmental Health Sciences | <https://www.niehs.nih.gov/health/topics/agents/pfc/index.cfm> |
| 16 | 12 | Vermont Department of Health | <https://www.healthvermont.gov/environment/drinking-water/perfluoroalkyl-and-polyfluoroalkyl-substances-pfas-drinking-water> |
| 17 | 13 | Michigan PFAS Action Response Team | <https://www.michigan.gov/pfasresponse/faq/categories/pfas-101> |
| 18 | 14 | CDC ATSDR | <https://www.atsdr.cdc.gov/pfas/health-effects/overview.html> |
| 19 | 15 | Massachusetts Department of Environmental Protection | <https://www.mass.gov/info-details/per-and-polyfluoroalkyl-substances-pfas-in-drinking-water> |
| 20 | 16 | Washington Department of Health | <https://doh.wa.gov/community-and-environment/contaminants/pfas> |
| 21 | 17 | Michigan PFAS Action Response Team | <https://www.michigan.gov/pfasresponse/drinking-water> |
| 22 | 18 | California Water Resources Control Board | <https://www.waterboards.ca.gov/drinking_water/certlic/drinkingwater/pfas.html> |
| 23 | 19 | Minnesota Department of Health | <https://www.health.state.mn.us/communities/environment/hazardous/topics/pfcs.html> |
| 24 | 20 | North Carolina Department of Environmental Quality | <https://www.deq.nc.gov/news/key-issues/emerging-compounds/understanding-pfas> |
| 25 | 21 | White House briefing | https://www.whitehouse.gov/briefing-room/statements-releases/2023/03/14/fact-sheet-biden-harris-administration-takes-new-action-to-protect-communities-from-pfas-pollution/ |
| 26 | 22 | National Geographic | <https://www.nationalgeographic.com/science/article/pfas-contamination-safe-drinking-water-study> |
| 27 | 23 | Natural Resources Defense Council | <https://www.nrdc.org/stories/forever-chemicals-called-pfas-show-your-food-clothes-and-home> |
| 28 | 24 | CNN | <https://www.cnn.com/2023/07/05/health/pfas-nearly-half-us-tap-water-wellness/index.html> |
| 29 | 25 | Vermont Department of Environmental Conservation | <http://dec.vermont.gov/water/drinking-water/water-quality-monitoring/pfas> |
| 30 | 26 | South Carolina Department of Health | <https://scdhec.gov/environment/polyfluoroalkyl-substances-pfas/pfas-bureau-water> |
| 31 | 27 | Wisconsin Department of Natural Resources | <https://dnr.wisconsin.gov/topic/PFAS/WaterQuality.html> |
| 32 | 28 | Oregon Health Authority | <https://www.oregon.gov/oha/ph/healthyenvironments/drinkingwater/operations/pages/pfas.aspx> |
| 33 | 29 | Montana Department of Environmental Quality | <https://deq.mt.gov/cleanupandrec/Programs/pfas> |
| 34 | 30 | Lake County Public Works | <https://www.lakecountyil.gov/4721/PFAS-and-Your-Water-Supply> |
| 35 | 31 | Connecticut Department of Public Health | <https://portal.ct.gov/DPH/Drinking-Water/DWS/Per--and-Polyfluoroalkyl-Substances> |
| 36 | 32 | Federal Drug Administration | <https://www.fda.gov/food/environmental-contaminants-food/and-polyfluoroalkyl-substances-pfas> |
| 37 | 33 | Indiana Department of Environmental Management | https://www.in.gov/idem/resources/nonrule-policies/per-and-polyfluoroalkyl-substances-pfas/ |
| 38 | 34 | Maine Division of Environmental and Community Health | <https://www.maine.gov/dhhs/mecdc/environmental-health/dwp/pws/pfas.shtml> |
| 39 | 35 | Safer States | https://www.saferstates.com/toxic-chemicals/pfas/  https://www.ocwd.com/what-we-do/water-quality/pfas/ |
| 40 | 36 | Orange County Water District | https://www.ocwd.com/what-we-do/water-quality/pfas/ |
| 41 | 37 | Government Accountability Office | <https://www.gao.gov/products/gao-22-105135> |
| 42 | 38 | New Jersey Department of Health | <https://www.nj.gov/health/ceohs/documents/pfas_drinking%20water.pdf> |
| 43 | 39 | Tennessee Department of Environment and Conservation | <https://www.tn.gov/environment/policy/pfas.html> |
| 44 | 40 | Maryland Department of the Environment | <https://mde.maryland.gov/PublicHealth/Pages/PFAS-Landing-Page.aspx> |
| 45 | 41 | Pennsylvania Department of Environmental Protection | <https://www.dep.pa.gov/Citizens/My-Water/drinking_water/PFAS/Pages/default.aspx> |
| 46 | 42 | City of Ann Arbor Michigan | <https://www.a2gov.org/departments/water-treatment/Pages/PFAS-Information.aspx> |
| 47 | 43 | Valley Water | <https://www.valleywater.org/accordion/pfas> |
| 48 | 44 | City of San Jose | <https://www.sanjoseca.gov/your-government/departments-offices/environmental-services/water-utilities/drinking-water/water-quality/pfas> |
| 49 | 45 | Veterans Affairs Public Health | <https://www.publichealth.va.gov/exposures/pfas.asp> |
| 50 | 46 | Silent Spring Institute | https://pfas-exchange.org/pfas-and-drinking-water-what-you-should-know/ |
| 51 | 47 | Idaho Department of Environmental Quality | https://www.deq.idaho.gov/water-quality/drinking-water/pfas-and-idaho-drinking-water/ |
| 52 | 48 | Clean Water | <https://cleanwater.org/pfas-chemicals-protecting-our-drinking-water-and-our-health> |
| 53 | 49 | Utah Department of Environmental Quality | <https://deq.utah.gov/drinking-water/drinking-water-pfas> |
| 54 | 50 | WAlaska Department of Environment Conservationaters Corporation | <https://www.waters.com/nextgen/us/en/applications/environmental/pfas-analysis-solutions.html> |
| 55 | 51 | Tampa Bay Water | https://www.tampabaywater.org/quality/water-quality-concerns/pfas/ |
| 56 | 52 | World Health Organization | <https://www.who.int/teams/environment-climate-change-and-health/water-sanitation-and-health/chemical-hazards-in-drinking-water/per-and-polyfluoroalkyl-substances> |
| 57 | 53 | City of Newport Beach | <https://www.newportbeachca.gov/government/departments/utilities/water-services/pfas-pfoa-and-drinking-water> |
| 58 | 54 | Philadelphia Water Department | https://water.phila.gov/sustainability/watershed-protection/pfas/ |
| 59 | 55 | DC Water | <https://www.dcwater.com/pfas-and-drinking-water> |
| 60 | 56 | Prince William County Water | <https://www.pwcsa.org/pfas-drinking-water-faq> |
| 61 | 57 | New York State Dept. of Environmental Conservation | <https://www.dec.ny.gov/chemical/108831.html> |
| 62 | 58 | Kentucky Energy and Environmental Cabinet | <https://eec.ky.gov/Environmental-Protection/Water/Protection/Pages/PFAS.aspx> |
| 63 | 59 | Hawaii Department of Health | https://health.hawaii.gov/heer/environmental-health/highlighted-projects/pfas/ |
| 64 | 60 | Alaska Department of Environment Conservation | https://dec.alaska.gov/spar/csp/pfas/ |
| 65 | 61 | Water Environment Federation | <https://www.wef.org/pfas> |
| 66 | 62 | Alameda County | <https://www.acwd.org/734/Understanding-PFAS> |
| 67 | 63 | American Water Works Association | <https://www.awwa.org/Resources-Tools/Resource-Topics/PFAS> |
| 68 | 64 | Scientific American | https://www.scientificamerican.com/article/forever-chemicals-are-widespread-in-u-s-drinking-water/ |
| 69 | 65 | National Science Foundation | <https://www.nsf.org/blog/consumer/treat-pfas-drinking-water> |
| 70 | 66 | Department of Defense | https://www.acq.osd.mil/eie/eer/ecc/pfas/ |
| 71 | 67 | Massachusetts Water Resource Authority | <https://www.mwra.com/watertesting/pfas/about.html> |
| 72 | 68 | Fairfax Water | <https://www.fairfaxwater.org/water-quality/facts-about-pfas> |
| 73 | 69 | Denver Water | <https://www.denverwater.org/your-water/water-quality/pfas> |
| 74 | 70 | Mississippi Department of Environmental Quality | https://www.mdeq.ms.gov/water/groundwater-assessment-and-remediation/pfas-information/ |
| 75 | 71 | Michigan League of Conservation Voters | https://michiganlcv.org/pfas/ |
| 76 | 72 | The Journalist's Resource | https://journalistsresource.org/home/pfas-forever-chemicals-in-drinking-water-an-explainer-and-research-roundup/ |
| 77 | 73 | National Conference of State Legislatures | <https://www.ncsl.org/environment-and-natural-resources/per-and-polyfluoroalkyl-substances> |
| 78 | 74 | The Water Research Foundation | <https://www.waterrf.org/research/topics/and-polyfluoroalkyl-substances-pfas> |
| 79 | 75 | New Orleans Sewage and Water Board | <https://www.swbno.org/DrinkingWater/PFAS> |
| 80 | 76 | University of Rochester Medical Center | <https://www.urmc.rochester.edu/news/story/pfas-what-you-need-to-know> |
| 81 | 77 | City of Sugar Land | <https://www.sugarlandtx.gov/2461/PFAS> |
| 82 | 78 | RTI International | <https://www.rti.org/focus-area/pfas-in-water> |
| 83 | 79 | Livingston New Jersey | <https://www.livingstonnj.org/1484/PFOA-PFAS-Important-Information-About-Dr> |
| 84 | 80 | Environmental International article | <https://www.sciencedirect.com/science/article/pii/S0160412023003069> |
| 85 | 81 | City of Rockford | <https://www.rockfordil.gov/315/PFAS-Drinking-Water> |
| 86 | 82 | Harvard School of Public Health | https://www.hsph.harvard.edu/news/press-releases/communities-of-color-disproportionately-exposed-to-pfas-pollution-in-drinking-water/ |
| 87 | 83 | Georgia Environmental Protection Division | <https://epd.georgia.gov/pfoa-and-pfos-information> |
| 88 | 84 | Virginia Department of Health | https://www.vdh.virginia.gov/drinking-water/pfas/ |
| 89 | 85 | Kansas Division of the Environment | <https://www.kdhe.ks.gov/635/Per--Polyfluoroalkyl-Substances> |
| 90 | 86 | City of Cincinnati | https://www.cincinnati-oh.gov/water/water-quality-and-treatment/water-your-health/pfas/ |
| 91 | 87 | Colorado Department of Public Health and Environment | <https://cdphe.colorado.gov/pfas-water> |
| 92 | 88 | City of Dayton | <https://www.daytonohio.gov/775/PFAS-and-Drinking-Water> |
| 93 | 89 | Seattle Public Utilities | <https://www.seattle.gov/utilities/your-services/water/water-quality/quality-concerns/pfas> |
| 94 | 90 | City of Sacramento Utilities | <https://www.cityofsacramento.org/Utilities/Water/Water-Quality/water-quality/PFAS> |
| 95 | 91 | City of Nashville | <https://www.nashville.gov/departments/water/water-quality/pfas> |
| 96 | 92 | Association of State Drinking Water Administration | https://www.asdwa.org/pfas/ |
| 97 | 93 | Missouri Department of Natural Resources | <https://dnr.mo.gov/contaminant-spotlight/perfluoroalkyl-polyfluoroalkyl-substances-pfas> |
| 98 | 94 | City of Milwaukee | <https://city.milwaukee.gov/water/WaterQuality/PFAS> |
| 99 | 95 | City of Newport | <https://www.nnva.gov/2754/PFAS> |
| 100 | 96 | Delaware Department of Natural Resources | https://dnrec.alpha.delaware.gov/waste-hazardous/remediation/pfas/ |
| 101 | 97 | WSSC Water | <https://www.wsscwater.com/pfas> |
| 102 | 98 | ABC News | <https://abcnews.go.com/US/pfas-entering-americas-water-supply/story?id=98479678> |
| 103 | 99 | Agilent | <https://www.agilent.com/en/solutions/environmental/water-testing/pfas-in-water> |
| 104 | 100 | Washington Post | https://www.washingtonpost.com/wellness/2023/06/24/pfas-water-forever-chemicals/ |

**2. APPENDIX B**

**Table B.1**

*Unstandardized Means and Standard Deviations by Source Type*

|  | **Scale** | **Federal** | | **State** | | **Local** | |  |
| --- | --- | --- | --- | --- | --- | --- | --- | --- |
|  |  | **M** | **SD** | **M** | **SD** | **M** | **SD** |  |
| Risk | 1-7 | 2.96 | 1.46 | 2.81 | 1.43 | 2.32 | 1.20 |  |
| Susceptibility | 1-7 | 3.83 | 1.67 | 3.49 | 1.61 | 2.68 | 1.43 |  |
| Coder-rated Self-efficacy | 1-7 | 1.29 | 1.68 | 2.13 | 1.65 | 1.21 | 1.52 |  |
| Collective Efficacy | 1-7 | 3.33 | 1.50 | 3.21 | 1.44 | 3.89 | 1.30 |  |
| Positive Emotion | 0-100% | .007 | .02 | .04 | .09 | .04 | .08 |  |
| Negative Emotion | 0-100% | .004 | .01 | .04 | .11 | .02 | .04 |  |
| Anxiety | 0-100% | .004 | .01 | .01 | .06 | 0 | 0 |  |
| LIWC Efficacy | 0-100% | 1.10 | .84 | 1.25 | 1.03 | 1.59 | .81 |  |
|  | **Water Utility** | | **Nonprofit** | | **News Media** | | **Research** | |
|  | **M** | **SD** | **M** | **SD** | **M** | **SD** | **M** | **SD** |
| Risk | 2 | 1.23 | 3.14 | 1.40 | 4.45 | 1.28 | 4.17 | 1.44 |
| Susceptibility | 2.91 | 1.40 | 3.59 | 1.61 | 5.18 | 1.45 | 4.58 | 1.59 |
| Coder-rated Self-efficacy | 2.5 | 1.56 | 1.91 | 1.53 | 3.45 | 1.62 | 2.67 | 1.56 |
| Collective Efficacy | 3.64 | 1.30 | 4.23 | 1.42 | 4.36 | 1.23 | 4.17 | 1.42 |
| Positive Emotion | .01 | .05 | .03 | .05 | .13 | .05 | .04 | .07 |
| Negative Emotion | .05 | .10 | .07 | .07 | .25 | .25 | .03 | .04 |
| Anxiety | .02 | .06 | .03 | .05 | .19 | .23 | .03 | .05 |
| LIWC Efficacy | 1.99 | .82 | 1.49 | .41 | 1.96 | 1.33 | 1.96 | 2.51 |
